# Enhancement of the Water Affinity of Histidine by Zinc and Copper Ions

**DOI:** 10.3390/ijms23073957

**Published:** 2022-04-02

**Authors:** Yongshun Song, Jing Zhan, Minyue Li, Hongwei Zhao, Guosheng Shi, Minghong Wu, Haiping Fang

**Affiliations:** 1 School of Physics, East China University of Science and Technology, Shanghai 200237, China; songyongshun11@mails.ucas.ac.cn; 2 Shanghai Applied Radiation Institute, Shanghai University, Shanghai 200444, China; zhanjing0215@163.com (J.Z.); 18800313926@163.com (M.L.); gsshi@shu.edu.cn (G.S.); 3 Zhangjiang Laboratory, Shanghai Advanced Research Institute, Chinese Academy of Sciences, Shanghai 201210, China; zhaohongwei@zjlab.org.cn; 4 Shanghai Institute of Applied Physics, Chinese Academy of Sciences, Shanghai 201800, China; 5 Wenzhou Institute, University of Chinese Academy of Sciences, Wenzhou 325000, China

**Keywords:** solubility, aromatic amino acids, cation–π interaction, transition-metal ions

## Abstract

Histidine (His) is widely involved in the structure and function of biomolecules. Transition-metal ions, such as Zn^2+^ and Cu^2+^, widely exist in biological environments, and they are crucial to many life-sustaining physiological processes. Herein, by employing density function calculations, we theoretically show that the water affinity of His can be enhanced by the strong cation–π interaction between His and Zn^2+^ and Cu^2+^. Further, the solubility of His is experimentally demonstrated to be greatly enhanced in ZnCl_2_ and CuCl_2_ solutions. The existence of cation–π interaction is demonstrated by fluorescence, ultraviolet (UV) spectroscopy and nuclear magnetic resonance (NMR) experiments. These findings are of great importance for the bioavailability of aromatic drugs and provide new insight for understanding the physiological functions of transition metal ions.

## 1. Introduction

Histidine (His) is one of the most essential and naturally occurring aromatic amino acids. As one of the building blocks of proteins, it is commonly involved in the structure and function of proteins, since its side-chain contains an imidazole ring which can easily participate in π–π [1,2], hydrogen bond [3,4], coordinate bond [5,6,7,8] and cation–π interactions [9,10,11]. For example, the coordination of ligand to the imidazole ring of His can inhibit the enzymic activity of tyrosine hydroxylase [12] and π–π stacking between His and Tyr is associated with the mitogenicity function of lectin [13]. Besides, histidine-rich peptides can be incorporated into polymers, liposomes, and proteins, including virus-like particles [14,15,16].

In many proteins, His is coordinated with transition metal ions, among which zinc and copper are the two with the most occurrence. This coordination is critical to the structures and functions of biomolecules [17,18,19,20,21]. For instance, the carboxylate–histidine–zinc triad is frequently found in zinc proteins and is important in the function of these metalloproteins [22]. The riboflavin binding protein (RBP) can bind copper and form a distinct well-ordered type II site under dialysis conditions [19]. In addition, the zinc–histidine complex had been used as a zinc supplement and was shown better absorption than zinc sulfate in humans [23,24]. The copper–histidine complex has been found in human blood, and has been applied to treat Menkes disease [25].

Most biochemical processes occur in aqueous solution. The dispersion behavior of biomolecules in water is important for their participation in physical, chemical, and biological processes [26,27,28,29,30]. Previous studies on the coordination of His with transition metal ions are mainly focused on the specific coordination modes and related biological functions. Whereas, studies on some general features of this coordination, such as the hydrophilicity of His, are fewer. By the binding of zinc or copper, the hydrophilicity of His will be modified, which will have significance for a wide range of problems, such as protein folding [31,32], structure stabilization [33,34]. The effect on the hydrophilicity of His by the binding of zinc/copper can be studied through the solubility change for His and complex Zn^2+^/Cu^2+^-His. Shi et al. studied the solubility of tryptophan (Trp) in transition metal ion salt solutions and found that the solubility of Trp is dramatically increased [35].

In this paper, by employing DFT calculations, we investigate the interaction between His and transition metal ions Zn^2+^ and Cu^2+^. Experiments on the solubility of His in ZnCl_2_ and CuCl_2_ solutions were then performed to verify the results of theoretical calculations. First principles calculations and solubility experiments strongly support that the strong cation–π interaction between His and transition metal ions Zn^2+^ and Cu^2+^ greatly affects the water affinity of His. Finally, the cation–π interaction between His and Zn^2+^/Cu^2+^ was demonstrated by fluorescence, ultraviolet (UV) spectroscopy and nuclear magnetic resonance (NMR) experiments.

## 2. Results and Discussion

**Figure 1:**
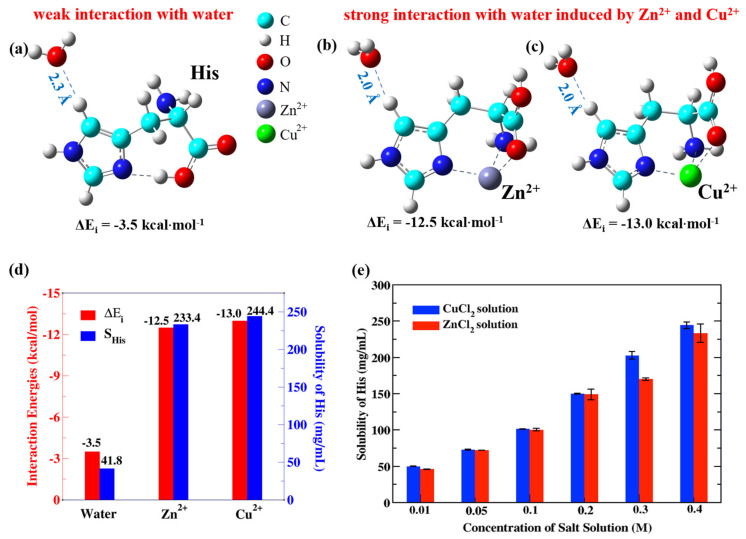
Interaction energies between His, Zn^2+^–His, Cu^2+^–His and the nearest water and the solubilities of His in ZnCl_2_ and CuCl_2_ solutions. (**a**–**c**) Optimized geometric structures of the His–water, Zn^2+^–His–water, and Cu^2+^–His–water systems and the interaction energies between the imidazole rings and the nearest water molecules. (**a**) Optimized distance between the imidazole ring in His and the nearest water molecule is 2.3 Å, and the interaction energy between them is −3.5 kcal·mol^−1^. (**b**) Optimized distance between the imidazole ring and the nearest water molecule after Zn^2+^ binding decreases to 2.0 Å, and the interaction energy increases to −12.5 kcal·mol^−1^. (**c**) Optimized distance between the imidazole ring in His and the nearest water molecule after Cu^2+^ binding decreases to 2.0 Å, and the interaction energy increases to −13.0 kcal·mol^−1^. (**d**) Interaction energies (ΔE_i_) between the imidazole ring in His and the nearest water molecule, without binding to any metal ion, with Zn^2+^ binding, and with Cu^2+^ binding and the solubilities of His (S_His_) in pure water, 0.4 M ZnCl_2_ solution, and 0.4 M CuCl_2_ solution. (**e**) Solubilities of His in CuCl_2_ and ZnCl_2_ solutions.

To investigate the interaction between His and transition metal ions Zn^2+^ and Cu^2+^, we first calculated the interaction energies between the imidazole ring in His with and without Zn^2+^ binding and the nearest neighbor water molecule by density functional theory (DFT). As shown in Figure 1b, when Zn^2+^ interacts with His, the interaction energy between the imidazole ring in His and the nearest water molecule is about −12.5 kcal·mol^−1^, which is much larger than the corresponding interaction energy without Zn^2+^ binding (−3.5 kcal·mol^−1^, Figure 1a). In addition, we also calculated the interaction energy between the imidazole ring in His and the nearest water molecule with Cu^2+^ binding, which is about −13.0 kcal·mol^−1^ (Figure 1c). These results show that both Zn^2+^ and Cu^2+^ greatly increase the water affinity of His to about the same degree.

It should be pointed out that in the most stable structures of Zn^2+^–His and Cu^2+^–His, Zn^2+^ and Cu^2+^ interact with the side of the imidazole ring together with the amino N atom and carbonyl O atom rather than at the top of the imidazole ring. The interaction energy of this ring/N/O tridentate coordination is 20.3 kcal·mol^−1^ larger than that of the structure in which Zn^2+^ stays at the top of the imidazole ring (Figure S1). Rimola et al. also pointed out that the configuration of Cu^2+^−His with Cu^2+^ at the top of the imidazole ring was not obtainable by first principle calculations [36]. Besides, Sastry et al. calculated the interaction between multiple cations and imidazole, finding that cations show affinity only toward the heteroatom N atom instead of the whole π–face of the imidazole ring [9]. Thus, the configuration of Zn^2+^/Cu^2+^ stays at the top of the imidazole ring, which interacts with a traditional cation–π interaction way, could not be the real complex in the experiments. For the configuration of ring/N/O tridentate coordination, it is expected that there is also a cation–π interaction between Zn^2+^/Cu^2+^ and His, because the π electron of imidazole ring and valence electron of Zn^2+^/Cu^2+^ are both involved in the HOMO-1 orbital (Figure S2) and the electron distribution of the whole imidazole ring is affected by the Zn^2+^ binding (Figure S3). To further analyze the cation−π interactions, we provide EDA calculations on the Zn^2+^/Cu^2+^−His complex. EDA can divide the total interaction energy into several interaction terms with physical meaning and is widely used for analyzing various intermolecular interactions [37,38]. We divided the optimized structure of Zn^2+^/Cu^2+^−His into two parts, i.e., the Zn^2+^/Cu^2+^ ion and His, and then performed single-point calculations at the B3LYP/TZP level of theory in the framework of DFT by using the ADF program [39]. The total interaction energies between Zn^2+^/Cu^2+^ and His were decomposed into Pauli repulsion, orbital interaction and electrostatic interaction (Table S1). The EDA results show that the electrostatic interactions and orbital interactions approximately equally contribute to the interaction energy, and the Pauli repulsion affords the main repulsive energy. 

We also would like to note that the geometric structures of the Zn^2+^–His–water and Cu^2+^–His–water systems were optimized based on the water molecule forming a hydrogen bond with the imidazole ring at the C–H site (Figure 1b,c). In fact, water can interact with the imidazole ring of Zn^2+^–His and Cu^2+^–His complexes at other sites, for example, N−H of the imidazole ring (shown in Figure S4), which also indicate that the water affinity of imidazole ring is increased. Since N−H is not as hydrophobic as C−H in His, the increase of water affinity of N−H is not as critical as C−H for the water affinity enhancement of the whole complex.

The water affinity is well related to solubility. A weak interaction with water indicates that hydration of the compound is unfavorable, contributing to poor water solubility. Because the water affinities of Zn^2+^–His and Cu^2+^–His are much larger than that of His, we expected that the solubilities of His in ZnCl_2_ and CuCl_2_ solutions would both dramatically increase. We then performed experiments on the solubilities of His (*S*_His_) in ZnCl_2_ and CuCl_2_ aqueous solutions. The solubility of His in ZnCl_2_ solution increases as much as that in CuCl_2_ solution (Figure 1d). In 0.4 M ZnCl_2_ and CuCl_2_ solutions, the solubility of His can increase to more than 5 times that in pure water. This is consistent with the first principles calculations of the interaction energy between the aromatic ring structure in His and the nearest water molecule. The results of His were then compared with previous work on Trp to show the enhanced water affinity of His by Zn^2+^ is nontrivial. Previous work on Trp [35] showed that the solubility of Trp in ZnCl_2_ solution slightly increases compared with the solubility in water (17.1 mg·mL^−1^ in 0.5 M ZnCl_2_ solution vs. 11.4 mg·mL^−1^ in pure water, Table 1). These results indicate that the aromatic amino acid His has a binding preference for Zn^2+^ compared with other amino acids and the interaction between them can greatly enhance the water affinity of His. 

We would like to point out that there is no obvious difference when different orders of adding cation ions and His are employed. Both orders will lead to the same result that the solubility of His is enhanced significantly. This is a major difference between His in this work and Trp in previous work [35], where the order of adding cations and Trp show totally different results for the solubility enhancement of Trp.

The solubility behavior of His with respect to the concentrations of ZnCl_2_ and CuCl_2_ solutions was then investigated. As shown in Figure 1e and Figure S5, the solubility behavior can be well fitted by $S_{A}=A_{M}C_{M}+S_{A}^{0}$, where $S_{A}$ and $S_{A}^{0}$ are the solubilities of amino acid *A* in the salt solution and pure water, $A_{M}$ is the water affinity factor of amino acid *A* induced by metal ion M^2+^, and $C_{M}$ is the concentration of M^2+^. $A_{M}$ has a distinct physical meaning that for every M salt concentration increase, the solubility of the amino acid increases by $A_{M}$ M. For His, $A_{\mathrm{Cu}}$ = 4.86 and $A_{\mathrm{Zn}}$ = 2.80, which are both much greater than the water affinity factor of Trp induced by Cu^2+^ ($A_{\mathrm{Cu}}$ = 0.46) [35].

It should be noted that different Zn^2+^–His and Cu^2+^–His complexes exist at different *p*H values in experiments [40]. The structure that we used for the DFT calculations was referred to as the M(HL)^2+^ species in refs [40,41]. Under weak acid conditions, this structure of tridentate coordination is the most accepted one [42,43]. Indeed, the *p*H values of the ZnCl_2_ and CuCl_2_ solutions used in the experiments correspond to the weak acid condition (5.24–5.75 for the ZnCl_2_ solutions and 3.04–5.75 for the CuCl_2_ solutions) (Tables S2 and S3).

To verify that the increase of the solubility mainly comes from the cation–π interaction between the transition-metal ion and the imidazole ring in His, the solubility of the non-aromatic amino acid glycine (Gly) was measured under the same conditions. In Figure S5, it can be found that the solubilities of Trp and His both dramatically increased in CuCl_2_ solutions, while the solubility of Gly only showed a slight increase. This result strongly supports that the enhanced solubility of His can be mainly attributed to the imidazole ring in His. Zn^2+^ is expected to show the same behavior.

**Figure 2:**
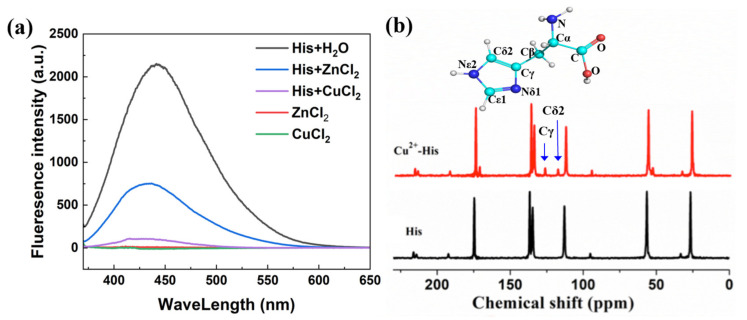
Fluorescence and NMR spectra on His in different solutions. (**a**) Fluorescence spectra of His in pure water, His in ZnCl_2_ solution, His in CuCl_2_ solutions (50 mM), ZnCl_2_ solution and CuCl_2_ solution. The concentrations of His in all the solutions are 30 mg·mL^−1^. The concentrations of ZnCl_2_ and CuCl_2_ are 50 mM in all the samples. (**b**) Cross–polarization/magic angle spinning (CP/MAS) NMR C12 spectra of Cu^2+^–His and the control group (His in pure water). The Cδ2 and Cγ peaks are labeled.

Fluorescence and ultraviolet (UV) absorption spectral experiments were then performed to provide evidence for the cation–π interaction between the metal ion and the imidazole ring in His. In Figure 2a, the fluorescence spectrum of His excited at 360 nm has an emission peak at 448 nm, which is assigned to the conjugated double bonds of the imidazole ring that can easily generate the π–π* transition [44]. Compared with the fluorescence intensity of His in water, the intensity of His in 50 mM CuCl_2_ solution markedly decreased, indicating that the conjugated double bonds of the imidazole ring in His were greatly affected in CuCl_2_ solution. The fluorescence spectrum of His in ZnCl_2_ solution is also shown in Figure 2a. Again, the fluorescence intensities of His and ZnCl_2_ were quenched when the Zn^2+^–His complex was formed, but to a smaller degree. The reason for the difference of the quenching degree in fluorescence is beyond the scope of this study. These results indicate that in ZnCl_2_ and CuCl_2_ solutions, Zn^2+^ and Cu^2+^ directly interact with the imidazole ring in His and decrease the fluorescence intensity of His. The imidazole ring of His exhibits absorption in the lower UV region (220 nm) [45]. Here, we observed that the UV absorption spectrum of His was also affected by the cation–π interaction between the imidazole ring in His and Cu^2+^ in solution (Figure S6), which is important evidence for the existence of the cation–π interaction. Overall, the fluorescence and UV absorption spectral experiments show the existence of the cation–π interaction between the imidazole ring in His and the metal ion, which is consistent with the theoretical prediction.

Nuclear magnetic resonance (NMR) experiments on C12 also showed that Cu^2+^–His had a clear chemical shift at 130 ppm, which is attributed to the imidazole ring in His (Figure 2b) [46]. Zn^2+^–His also showed a chemical shift at 130 ppm, although with a weaker signal (data not shown).

The structure of His did not change much after the binding of the transition metal ions Zn^2+^ and Cu^2+^. Infrared (IR) spectra of the dried samples of His in ZnCl_2_ and CuCl_2_ solutions were similar to that in pure water (Figure S7) [47,48].

The enhancement of *S*_His_ in ZnCl_2_ and CuCl_2_ solutions does not come from the *p*H effect induced by hydrolysis of Zn^2+^ or Cu^2+^. As shown in Tables S2 and S3, the *p*H values of the ZnCl_2_ and CuCl_2_ solutions both slightly decreased (from 6.0 to 5.2 for Zn^2+^ and from 4.5 to 3.1 for Cu^2+^), indicating that the *p*H effect does not greatly contribute to the increased solubility of His.

## 3. Materials and Methods

### 3.1. Computational Methods

The B3LYP functional [49] in the framework of DFT is used to calculate the Zn^2+^–His–H_2_O and Cu^2+^–His–H_2_O systems with Gaussian 09 package [50]. Geometric structures were first optimized by Berny algorithm [51] with the convergence criteria of a maximum step size of 0.0018 au and a root mean square force of 0.0003 a.u. A hybrid pseudo potential LanL2DZ is employed to calculate Cu^2+^ and Zn^2+^, while other atoms are calculated at the 6–31+G(d,p) basis set level (see detailed methods in the Supplementary Material (SM)). 

Interaction energies between water molecule and aromatic ring of amino acid (AA) with and without metal ion (M) binding are represented as Δ_*Ei*_, which can be calculated as,

(1) $$\Delta E_{i}=E_{\mathrm{Total}}-E_{\mathrm{complex}}-E_{w},$$

where, $E_{\mathrm{Total}}$, $E_{\mathrm{complex}}$, and $E_{w}$ are the single-point energies of His–M^2+^–H_2_O (or His–H_2_O), complex His–M^2+^ (or His), and H_2_O, respectively.

All the calculations are performed in vacuum condition. There is no other water molecule and ion except the nearest water and the bound ion we considered explicitly.

### 3.2. Experiments Materials

L–Histidine (His, 99%) and Glycine (Gly, 99%) were purchased from Sigma-Aldrich, Shanghai, China. Zinc(II) chloride (ZnCl_2_, 98%) was purchased from J&K Scientific Ltd, Beijing, China. Copper(II) chloride dihydrate (CuCl_2_·2H_2_O) (99%) was purchased from Sinopharm Chemical Reagent Co., Ltd, Beijing, China. All samples were used without purification and preprocessing. All salt solutions and other aqueous solutions were prepared with 18.2 MΩ, 3 ppb TOC Milli–Q water (Millipore, Burlington, MA, USA).

### 3.3. Solubility Measurement

Histidine (or Glycine) powder was added into pure water (or into the given 0.01–0.4 M CuCl_2_ and ZnCl_2_ solutions) with constant shaking until apparent saturation (with some insoluble His (or Gly) powder appeared), and then the solution was kept continuously stirring for 24 h in a thermostat at 25 °C. Afterward, the solution was centrifuged at 25 °C to remove insoluble His (or Gly) powder. Then, approximately 1 mL of solution was withdrawn by a pipette from the supernatant phase and transferred to a clean and weighed centrifuge tube. These centrifuge tubes were then transferred to liquid nitrogen for freezing and lyophilized overnight in a vacuum flask at 0.125 mbar and −58 °C in a freeze-dryer (Virtis Freezer Dryer, New York, NY, USA). The drying process was repeated until a constant mass reading was achieved. The solubility of His (or Gly) was calculated by the mass value difference of the centrifuge tube after removing salt mass. The data reported in this work were ensured by measurement of solubility for at least three replicate experiments at all compositions (Tables S2 and S3). Based on our measurement strategy, the solubility of His in pure water is 41.9 mg·mL^−1^, consistent with previous reports [52].

### 3.4. Measurement of pH

The *p*H values were measured by SevenCompact™ S220 *p*H meter (METTLER TOLEDO, Zurich, Switzerland) (*p*H = 0~14).

### 3.5. UV Spectroscopy

UV absorption spectra of His, CuCl_2_, and His (CuCl_2_) solutions were recorded on a U–3100 spectrophotometer (Hitachi, Tokyo, Japan). The concentration for His used in this experiment is 30 mg·mL^−1^, for CuCl_2_ is 50 mM.

### 3.6. Fluorescence Spectrofluorophotometer

Excitation and photoluminescence (PL) spectra were measured with a Hitachi 7000 fluorescence spectrophotometer (Tokyo, Japan) and emission slit width of 10 mm was used to record fluorescence spectra, and the fluorescence spectra of the work were recorded with $\lambda_{ex}/\lambda_{em}$= 360 nm/440 nm. The thickness of all liquid sample cells is 10 mm.

### 3.7. IR Spectra

IR spectra from 4000 cm^−1^ to 500 cm^−1^ of His, Zn^2+^–His, and Cu^2+^–His powder were measured using a Bio-Rad FTIR spectrometer FTS165 (Benton, ME, USA) equipped with resolution of 4 cm^−1^. The drying His, Zn^2+^–His, and Cu^2+^–His powder were obtained by freezing and lyophilized from corresponding saturated solution.

### 3.8. Solid-State NMR Spectroscopy

Solid-state NMR experiments were carried out on a wide-bore Bruker AVANCE-600 spectrometer (14.1 T) and a Bruker DSX-400 spectrometer (Karlsruhe, Germany) on 4-mm triple-resonance MAS probes. The drying His, Zn^2+^–His, and Cu^2+^–His powders were obtained by freezing and lyophilized from corresponding saturated solutions.

## 4. Conclusions

In summary, by first principles calculations, we have shown that the cation–π interaction between Zn^2+^ and His is very strong, which can enhance the water affinity of His to a comparable extent to Cu^2+^. Theoretical studies showed that the strong cation–π interaction between Zn^2+^/Cu^2+^ and His is the key. This cation–π interaction modifies the electronic distribution of the imidazole ring in His and significantly enhances the water affinity of His. Further EDA analysis showed that this cation–π interaction is approximately contributed equally by electrostatic interactions and orbital interactions. We also performed solubility experiments, which showed that the solubilities of His in ZnCl_2_ and CuCl_2_ solutions can reach more than 5 times that of His in pure water.

The results highlight that the solubility enhancement of many imidazole derivatives by Zn^2+^ and Cu^2+^ may be a general phenomenon and needs to attract more attention in different research fields, such as drug chemistry and colloid chemistry. These findings will enrich the understanding of the interactions between metal ions and biomolecules, and provide new insight into the physiological functions of multivalent metal ions.

We also would like to note that, our previous study on the solubility of Trp showed that Zn^2+^ is relatively weak in affecting the hydrophobicity of Trp by cation–π interaction, which is about one third of the solubility enhancement of Cu^2+^ [35]. Combined with this study, we found that the cation–π interaction is sensitive to the specific interaction environment, providing a possible scheme for the selectivity mechanism of biomolecules. Actually, Tu et al. recently showed that the cation–π interaction is the origin of the selectivity mechanism of calcium and magnesium in phosphotyrosine, demonstrating the sensitivity of cation–π interaction to different ion species with identical charges [53]. Considering the common binding mode of zinc-histidine in biology, it is expected histidine will participate in the selectivity mechanism of transition metal ions.

## Figures and Tables

**Table 1:** Solubilities of His and Trp in pure water, ZnCl_2_ and CuCl_2_.

		Pure Water	ZnCl_2_	CuCl_2_
Solubility (mg·mL^−1^)	His	41.9	233.4 ^1^	244.4 ^1^
	Trp	11.4	17.1 ^2^	57.6 ^2^

^1^ Salt concentration 0.4 M. ^2^ Salt concentration 0.5 M.

## Data Availability

Not applicable.

## Supplementary Materials

The following supporting information can be downloaded at: https://www.mdpi.com/article/10.3390/ijms23073957/s1. Reference [54] is cited in Supplementary Materials.
