# Supplementary material for: Enhancement of the Water Affinity of Histidine by Zinc and Copper Ions
Source: Int J Mol Sci. 2022 Apr 2;23(7):3957. doi: 10.3390/ijms23073957 (PMC8999569; doi:10.3390/ijms23073957)
Supplement: Supplementary file 1 [file ijms-23-03957-s001.zip › ijms-1620212-supplementary-SFS.pdf]

Supporting Information for

Enhancement of the Water Affinity of Histidine by Zinc and Copper Ions

Yongshun Song[1*], Jing Zhan[2*], Minyue Li[2], Hongwei Zhao[3,4], Guosheng Shi[2], Minghong Wu[2,*], Haiping Fang[1,5,*]

[1]*Department of Physics, East China University of Science and Technology, Shanghai 200237, China*

[2]*Shanghai Applied Radiation Institute, Shanghai University, Shanghai 200444, China*

[3]*Zhangjiang Laboratory, Shanghai Advanced Research Institute, Chinese Academy of Sciences, Shanghai 201210, China*

[4]*Shanghai Institute of Applied Physics, Chinese Academy of Sciences, Shanghai 201800, China*

[5]*Wenzhou Institute, University of Chinese Academy of Sciences, Wenzhou 325000, China*

Context

PS1: Optimized geometric structures and interaction energies between Zn^{2+} and His

PS2: HOMO and HOMO-1 orbitals of Zn^{2+}–His complex

PS3: Electron distribution of His and Zn^{2+}–His complex

PS4: Energy decomposition analysis (EDA) on the cation-π interactions between Zn^{2+}/Cu^{2+} and His.

PS5: More optimized geometric structures and interaction energies between water molecule and His with and without Zn^{2+} adsorption

PS6: Solubilities of Trp, His, and Gly in $CuCl_2$ aqueous solution.

PS7: UV absorption spectra of His, $CuCl_2$ and Cu^{2+}–His

PS8: Spectra of the IR of His powder, Zn^{2+}–His and Cu^{2+}–His precipitates

PS9: Solubility measurement data

mhwu@mail.shu.edu.cn; fanghaiping@sinap.ac.cn

[*] These authors contributed equally to this work.

PS1: Optimized geometric structures and interaction energies between Zn^{2+} and His

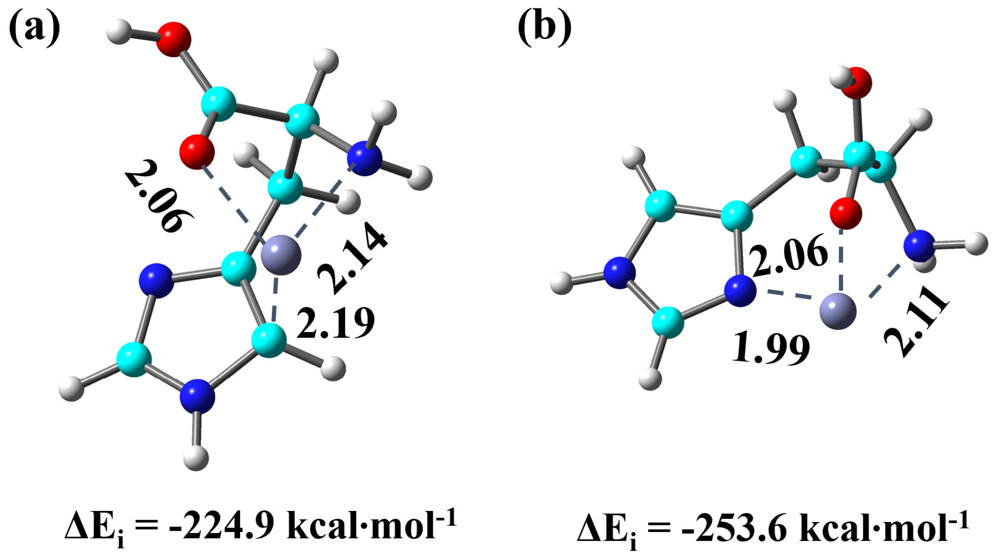

Figure S1. Two optimized geometric structures of Zn^{2+}–His. (a) Zn^{2+} binds to the imidazole ring in His together with amino N and the carbonyl O. The binding energy is –224.9 kcal·mol^{-1}. (b) Zn^{2+} binds to the imidazole N in His together with amino N and the carbonyl O. The binding energy is –253.6 kcal·mol^{-1}.

PS2: HOMO and HOMO-1 orbitals of Zn^{2+}–His complex

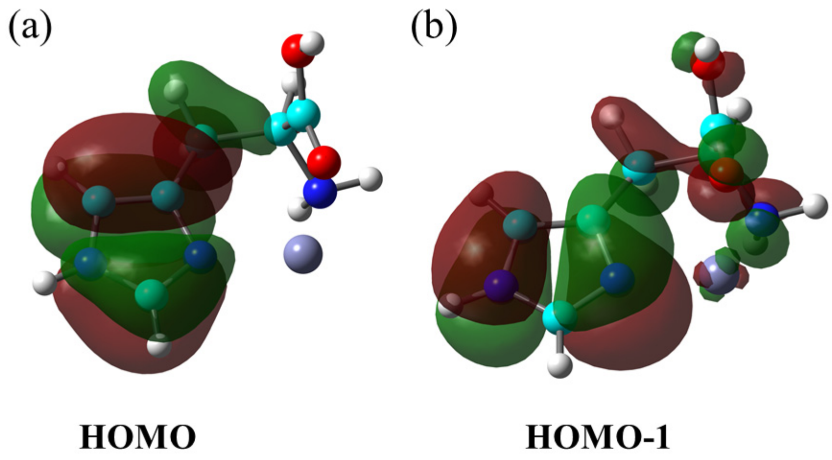

Figure S2. HOMO orbital (a) and HOMO-1 orbital (b) of Zn^{2+}–His with tridentate coordination configuration.

PS3: Electron distribution of His and Zn^{2+}–His complex

The charge values on atoms of His and Zn^{2+}–His are shown in Figure S3. It can be found that, more than one electron of His is transferred to Zn^{2+} after the adsorption of Zn^{2+}. Most of the electron transferred to Zn^{2+} comes from three regions: the NH_2 region, the COOH region, and the imidazole region. At the imidazole region, it is the N–H and two C–Hs that contributing most of the electron transferred, instead of the N atom nearest to Zn^{2+}. From this point, it clearly indicates that Zn^{2+} interacts with the π cloud of imidazole ring.

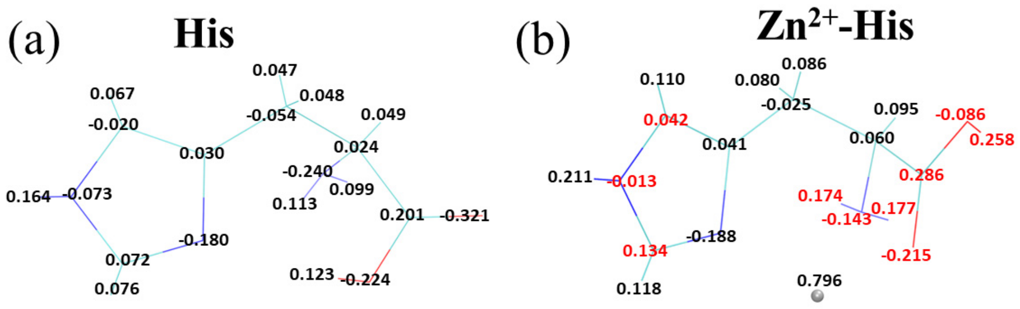

Figure S3. Hirshfeld charge distribution of His (a) and Zn^{2+}–His (b). The charge values that are at least 0.06e more positive than that in His is highlighted by red.

PS4: Energy decomposition analysis (EDA) on the cation-π interactions between Zn^{2+}/Cu^{2+} and His.

Table S1. EDA on the cation-π interactions between Zn^{2+}/Cu^{2+} and His by decomposing the optimized structures of Zn^{2+}/Cu^{2+}-His into two parts. The energy unit is kcal·mol^{-1}.

	Pauli repulsion	Orbital interaction	Electrostatic interaction	Total energy
Zn^{2+}-His	88.31	-184.03	-209.81	-305.53
Cu^{2+}-His	113.03	-221.84	-210.24	-319.05

PS5: More optimized geometric structures and interaction energies between water molecule and His with and without Zn^{2+} adsorption

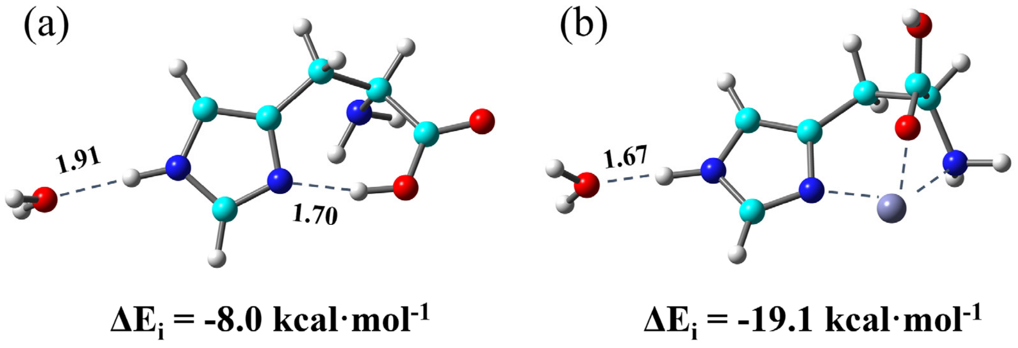

Figure S4. Optimized geometric structures of H_2O–His and H_2O–Zn^{2+}–His. (a) Water molecule binds to the amino N of imidazole ring in His. The binding energy is –8.0 kcal·mol^{-1}. (b) Water molecule binds to the amino N of imidazole ring in Zn^{2+}–His complex. The binding energy is –19.1 kcal·mol^{-1}.

PS6: Solubilities of Trp, His, and Gly in $CuCl_2$ aqueous solution.

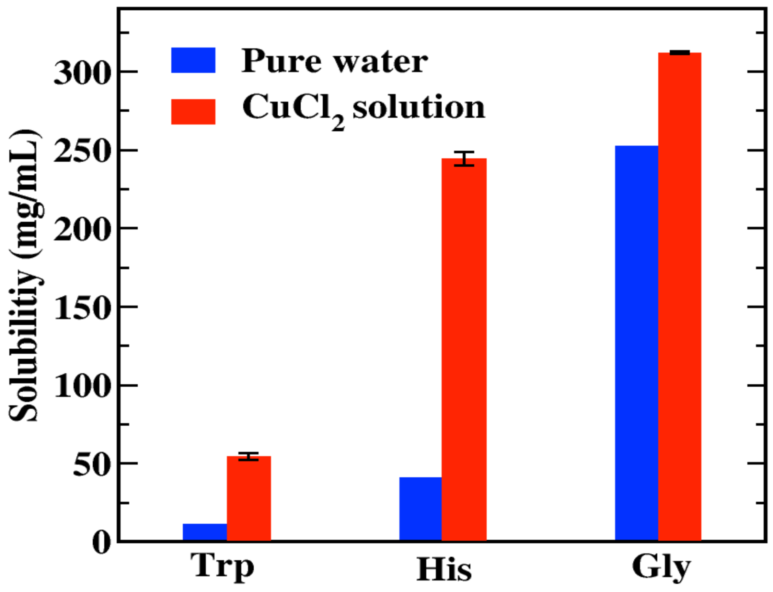

Figure S5. Solubilities of Trp, His, and Gly in pure water (blue bars) and 0.4 M $CuCl_2$ aqueous solution (red bars).

PS7: UV absorption spectra of His, $CuCl_2$ and Cu^{2+}–His

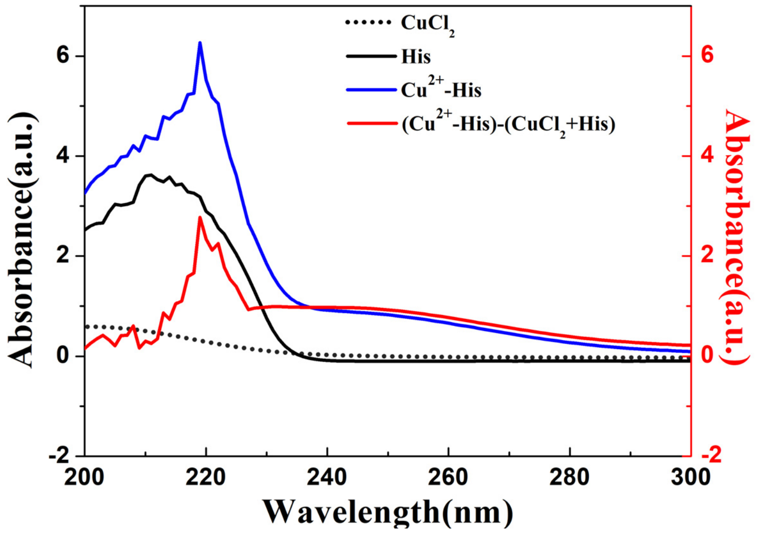

Figure S6. UV absorbance spectra of His (black solid line), $CuCl_2$ (black dotted line), Cu^{2+}–His (blue line) and the difference spectrum Cu^{2+}–His minus (His + $CuCl_2$) (red line).

PS8: Spectra of the IR of His powder, Zn^{2+}–His and Cu^{2+}–His precipitates

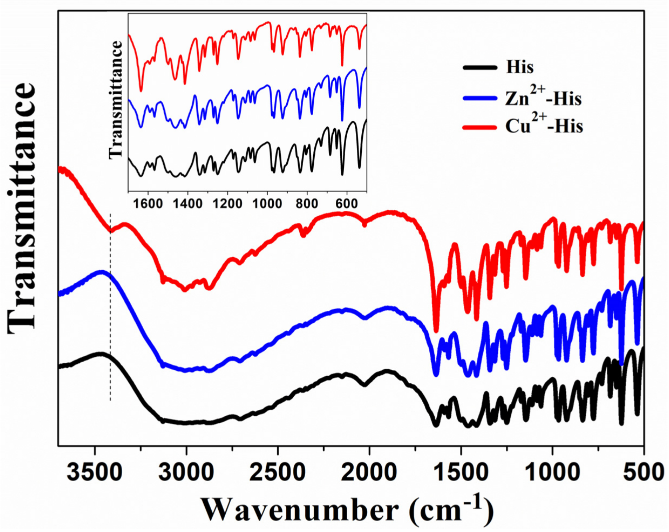

Figure S7. IR spectra of His powder, Zn^{2+}–His and Cu^{2+}–His precipitates.

PS9: Solubility measurement data

Table S2. The *p*H values of pure water, 0.01 M, 0.05 M, 0.1 M, 0.2 M, 0.3 M, and 0.4 M $ZnCl_2$ aqueous solutions, and the solubilities of the His in those solvents.

**Zn^{2+} concentrations （M）**	**Solubilities of His ($mg\cdot mL^{-1}$)**	***p*H**
0	41.07±0.32	5.75±0.04
0.01	46.07±0.17	5.89±0.48
0.05	72.18±0.23	5.82±0.46
0.1	100.42±1.87	5.65±0.43
0.2	148.96±7.67	5.58±0.11
0.3	170.07±1.80	5.40±0.18
0.4	233.41±12.80	5.24±0.22

Table S3. The *p*H values of pure water, 0.01 M, 0.05 M, 0.1 M, 0.2 M, 0.3 M, and 0.4 M $CuCl_2$ aqueous solutions, and the solubilities of His in those solvents.

**Cu^{2+} concentrations （M）**	**Solubilities of His ($mg\cdot mL^{-1}$)**	***p*H**
0	41.07±0.32	5.75±0.04
0.01	50.16±0.42	4.46±0.21
0.05	73.02±0.41	3.99±0.10
0.1	101.38±0.44	3.78±0.03
0.2	150.32±0.73	3.45±0.10
0.3	202.76±5.31	3.26±0.11
0.4	244.44±4.50	3.04±0.15

References:

1. Hirshfeld, F. L., Bonded-atom fragments for describing molecular charge densities. *Theor. Chim. Acta* **1977,** 44, (2), 129-138.
